# Human Mesenchymal Stem Cell Secretome Driven T Cell Immunomodulation Is IL-10 Dependent

**DOI:** 10.3390/ijms232113596

**Published:** 2022-11-06

**Authors:** Matthew T. Shephard, Marwan M. Merkhan, Nicholas R. Forsyth

**Affiliations:** 1 Guy Hilton Research Centre, School of Pharmacy and Bioengineering, Keele University, Staffordshire ST4 7QB, UK; 2 Department of Pharmacology and Toxicology, College of Pharmacy, University of Mosul, Mosul 41002, Iraq

**Keywords:** MSC, secretome, immunomodulation, physoxia

## Abstract

The Human Mesenchymal Stem Cell (hMSC) secretome has pleiotropic effects underpinning its therapeutic potential. hMSC serum-free conditioned media (SFCM) contains a variety of cytokines, with previous studies linking a changed secretome composition to physoxia. The Jurkat T cell model allowed the efficacy of SFCM vs. serum-free media (SFM) in the suppression of immunological aspects, including proliferation and polarisation, to be explored. Cell growth in SFM was higher [(21% O_2_ = 5.3 × 10^5^ ± 1.8 × 10^4^ cells/mL) and (2% O_2_ = 5.1 × 10^5^ ± 3.0 × 10^4^ cells/mL)], compared to SFCM [(21% O_2_ = 2.4 × 10^5^ ± 2.5 × 10^4^ cells/mL) and (2% O_2_ = 2.2 × 10^5^ ± 5.8 × 10^3^ cells/mL)]. SFM supported IL-2 release following activation [(21% O_2_ = 5305 ± 211 pg/mL) and (2% O_2_ = 5347 ± 327 pg/mL)] whereas SFCM suppressed IL-2 secretion [(21% O_2_ = 2461 ± 178 pg/mL) and (2% O_2_ = 1625 ± 159 pg/mL)]. Anti-inflammatory cytokines, namely IL-4, IL-10, and IL-13, which we previously confirmed as components of hMSC SFCM, were tested. IL-10 neutralisation in SFCM restored proliferation in both oxygen environments (SFM/SFCM^+antiIL−10^ ~1-fold increase). Conversely, IL-4/IL-13 neutralisation showed no proliferation restoration [(SFM/SFM^+antiIL−4^ ~2-fold decrease), and (SFM/SFCM^+antiIL−13^ ~2-fold decrease)]. Present findings indicate IL-10 played an immunosuppressive role by reducing IL-2 secretion. Identification of immunosuppressive components of the hMSC secretome and a mechanistic understanding of their action allow for the advancement and refinement of potential future cell-free therapies.

## 1. Introduction

Human Mesenchymal Stem Cells (hMSCs) are a promising therapeutic tool in regenerative medicine with the potential to treat a number of diseases and disorders [1,2]. However, the precise mechanisms of action remain unclear, though they are likely related to all or a combination of the following: multipotent differentiation, functional incorporation, immunomodulation, and secretion of paracrine factors [3,4]. hMSCs are of particular interest as a therapeutic tool for inflammatory diseases through their ability to suppress T cell proliferation [1,3,5,6,7]. The suppression has been shown to be a broad spectrum and involving mitogens, peptide antigens, alloantigen-induced T cell proliferation as well as Cluster of Differentiation 3 (CD3)/CD28 antibody mediated T cell activation [8,9,10]. Proteomic profiling of serum-free conditioned media (SFCM) from human MSCs (hMSCs) revealed the presence of a range of pleiotropic biomolecules within the secretome including the vascular endothelial growth factor (VEGF), granulocyte-macrophage colony-stimulating factor (GMCSF), and members of the interleukin (IL) family [11,12,13,14,15]. Additionally, MSCs suppress pharmacological activation of intracellular pathways of T cells, confirming that the mechanism of inhibition is a non-T-cell receptor-based pathway [16]. The suppression involves different T cell subtypes including both CD4+ and CD8+ as well as naïve T cells [4,17,18]. MSC-mediated immunosuppression was demonstrated by direct means including MSC interactions with T cells, plasma membrane proteins and through the release of soluble factors from MSCs [4]. Additionally, MSCs were shown to act through indirect mechanisms by the suppression of antigen-presenting cells. Pharmacological activation of T cells through the addition of Phorbol 12-myristate 13-acetate (PMA) or the antibiotic ionomycin to cell cultures was shown to act downstream of the T cell receptor complex. This is achieved through the activation of protein kinase C (PKC) by PMA or inducing a calcium ion influx by ionomycin. T cell proliferation stimulated by these factors is suppressed by MSCs, suggesting that the T cell receptor complex is not a target for the suppression and that MSCs exert their effects downstream of the protein kinase C and Ca^2+^ influx [19]. The suppression of T cells through utilization of the transwell cell culture system, where MSCs and lymphocytes are separated by a porous barrier membrane, further suggests that MSCs exert their immunosuppressive activity through paracrine signalling mechanisms [4]. In addition to in vitro evidence, in vivo T cell suppression by MSCs was confirmed in an experimental baboon animal model where the administration of MSCs prolonged the survival of a skin graft [5].

Upon activation, naïve T cells undergo polarisation with subsequent IL-2 production. The released IL-2 binds to an IL-2 cell surface receptor on the engaged T cell, inducing the mTOR pathway and resultant progression of the cell cycle and T cell proliferation [20,21]. During cell cycle progression, the localised cytokine microenvironment determines the fate of T cell differentiation into either Th1 or Th2 cells. For instance, the presence of the pro-inflammatory cytokine IL-12 in the surrounding vicinity promotes Th1 differentiation while IL-4 mediates Th2 differentiation [22]. Likewise, the presence of IL-10 in the T cell milieu promotes differentiation toward Treg [23]. Cytokine promotion of their action occurs through latent proteins involving Janus Kinase (JAK) and signal transducer and activator of transcription (STAT) [24]. Different anti-inflammatory cytokines, such as, IL-4, IL-10, and IL-12, use various post-receptor translation pathways to induce their effector function; STAT6, STAT3, and STAT4, respectively [25]. 

The role of oxygen in stem cell biology was described variously [26,27,28]. Physoxia is an inherent feature of the in vivo hMSC niche, drawing largely from the sinusoidal blood network characteristic of bone marrow [29,30,31]. Studies to define the pO2 of in vivo environments, specifically sinusoidal bone marrow, show an average value of 2.7% [32]. Physoxia is significantly lower than inhaled air (21% O_2_), and it declines gradually as it passes from the lung to the tissues, ranging between 0.1%–9% with an average of 2% O_2_ [31,33,34]. Applying an increasingly in-vivo-like physoxia to the in vitro hMSC culture modulates the transcriptome, and increasing evidence suggests this manifests itself via an altered secretome composition [35,36,37,38]. An altered secretome would likely impact the reparative action of SFCM and would likely better reflect the behaviour of hMSCs and/or their secretome following a transplant into in vivo tissues.

Jurkat, an immortalised acute leukemic cell line, is a standard surrogate for T cells [39,40]. Jurkat cells are polarised upon exposure to a combination of phorbol ester and a co-stimulator molecule, either ionomycin or phytohaemagglutinin (PHA), yielding a robust IL-2 release. PMA, a structural homologue for DAG, mediates its stimulation via activation of PKC resulting in the production of low amounts of IL-2 which is strongly accentuated by the addition of PHA. In the present study, the Jurkat cell line is used as a model to test the efficacy of SFCM in the suppression of proliferation and IL-2 secretion, both markers of Jurkat cell activation. In an attempt to identify the cytokine(s) mediating the immunosuppressive effect of SFCM, cytokines with prominent anti-inflammatory activity, namely, IL-4, IL-10, and IL-13, which were identified in our previously published study, were added individually to SFM or blocked from SFCM using their specific neutralising polyclonal antibody [15].

## 2. Results

### 2.1. Characterisation of In Vitro Jurkat T Cell Activation Model

#### 2.1.1. Morphological Assessment of Jurkat T Cells

**Figure 1:**
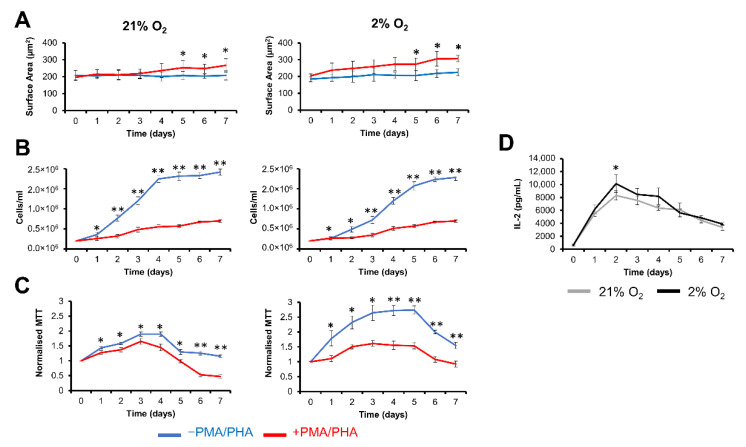
PMA/PHA stimulation results in suppression of proliferation and induction of polarisation. The proliferation and activation of Jurkat T cells were characterised in GM ^± PMA/PHA^ in both air (21% O_2_) and physoxia (2% O_2_) over 7 days. (**A**) Surface area measurements (μm^2^) of Jurkat T cells in GM ^± PMA/PHA^ over a 7 day period. Data are expressed as mean ± SD of 3 spots for each slide (*n* = 3). Statistical analysis consisting of a two-sample t-test was conducted to compare the two groups. (**B**) Proliferation was assessed using haemocytometer-based cell counting from 3 independent flasks (*n* = 3). (**C**) Corresponding optical density (O.D.) values for metabolic MTT assays on three replicate samples (*n* = 36). MTT assay values at each time point were normalised to day 0. (**D**) IL-2 concentration (pg/mL) in cell culture media in the presence of PMA/PHA inducers in both air and physoxia environments (*n* = 9). Statistical analysis consisting of a two-sample t-test was conducted to compare the different groups. Data expressed as mean ± SD, * *p* < 0.05, ** *p* < 0.001.

Jurkat T cells were activated through the addition of PMA and PHA in both air (21% O_2_) and physoxia (2% O_2_) environments over 7 days. Morphological assessment following polarisation established an increased cell surface area over 7 days (Figure 1A and Figure S1A,B). There was no significant difference between the polarised and non-polarised conditions at earlier timepoints; however, significant differences emerged at day 5 and thereafter in both oxygen tensions [21% O_2_ (GM^−PMA/PHA^ = 206 ± 24 µm^2^ and GM^+PMA/PHA^ = 253 ± 41 µm^2^) and 2% O_2_ (GM^−PMA/PHA^ = 206 ± 29 µm^2^ and GM^+PMA/PHA^ = 274 ± 37 µm^2^) (*p* < 0.05)].

#### 2.1.2. Proliferation and Polarisation of Jurkat T Cell

We next sought to determine if Jurkat activation had an effect on either proliferation or metabolic activity. In the absence of PMA/PHA, cell counts showed a trend of lag phase, log phase, and finally stationary phase (Figure 1B). In the presence of PMA/PHA, there was a significant (*p* < 0.001) reduction in proliferation at all time points, particularly at the start of the polarisation phase at day 2, GM^−PMA/PHA^ [21% O_2_ (7.7 × 10^5^ ± 7.6 × 10^4^ cells/mL)] and 2% O_2_ (4.9 × 10^5^ ± 6.3 × 10^4^ cells/mL)] versus GM^+PMA/PHA^ [21% O_2_ (3.2 × 10^5^ ± 4.4 × 10^4^ cells/mL)] and 2% O_2_ (2.8 × 10^5^ ± 2.1 × 10^4^ cells/mL)]. A significantly lower mitochondrial metabolism was observed in the presence of PMA/PHA compared to the control non-treated group (Figure 1C). In the absence of PMA/PHA, the cell metabolism was upregulated at day 3 [21% O_2_ (1.9 ± 0.1 O.D.) and 2% O_2_ (2.7 ± 0.3 O.D.)] followed by a decline (*p* < 0.05) thereafter reaching a minimum value at day 7 [21% O_2_ (1.2 ± 0.1 O.D.) and 2% O_2_ (1.6 ± 0.1 O.D.)]. In contrast, the addition of PMA/PHA was associated with a reduction in the metabolism at day 3 [21% O_2_ (1.7 ± 0.1 O.D.) and 2% O_2_ (1.6 ± 0.1 O.D.)]. Moreover, the polarisation of Jurkat T cells resulted in the production or release of IL-2 over 7 days reaching a maximum at day 2, which was significantly increased in 2% O_2_ compared to 21% O_2_ [21% O_2_ (8.3 × 10^3^ ± 707 pg/mL) and 2% O_2_ (1.0 × 10^4^ ± 1398 pg/mL) (*p* < 0.05)] (Figure 1D).

### 2.2. SFCM Protects Jurkat T Cells from PMA/PHA-Induced Morphological Changes

**Figure 2:**
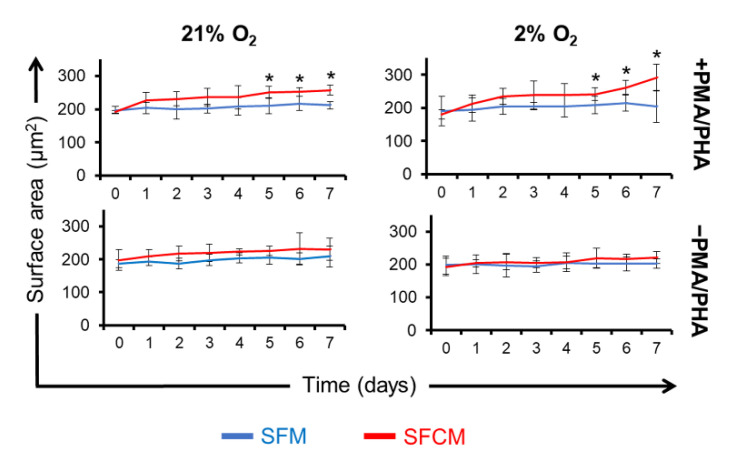
SFCM protects Jurkat T cells from PMA/PHA-induced morphological changes. Cell surface area measurements (μm^2^) of Jurkat T cells ± PMA/PHA cultured in SFM versus SFCM in both air (21% O_2_) and physoxia (2% O_2_) over 7 days. Jurkat T cell timepoint samples were cytospinned, fixed, and stained with Giemsa–May–Grunwald’s stain. Data expressed as mean ± SD of 3 spots for each slide (*n* = 3), * *p* < 0.05.

The Jurkat T cell surface area increased with the addition of PMA/PHA over 7 days in SFM with significance emerging from day 5 onwards [21% O_2_ (SFM^−PMA/PHA^ = 210 ± 23 µm^2^ and SFM^+PMA/PHA^ = 250 ± 18 µm^2^) and 2% O_2_ (SFM^−PMA/PHA^ = 208 ± 27 µm^2^ and SFM^+PMA/PHA^ = 241 ± 19 µm^2^) (*p* < 0.05)] in both oxygen tensions (Figure 2). Differences were minimised in SFCM showing no significant differences between polarised versus non-polarised culture conditions at day 5 [21% O_2_ (SFCM^−PMA/PHA^ = 206 ± 21 µm^2^ and SFCM^+PMA/PHA^ = 225 ± 14 µm^2^) and 2% O_2_ (SFCM^−PMA/PHA^ = 203 ± 13 µm^2^ and SFCM^+PMA/PHA^ = 218 ± 30 µm^2^) (*p* > 0.05)]. These results suggest that SFCM inhibited the increased surface area associated with activation by PMA/PHA.

### 2.3. A Role for IL-10 in SFCM-Induced Immunosuppression

#### 2.3.1. IL-10 Suppresses the Proliferation of Non-Polarised Jurkat T Cells

**Figure 3:**
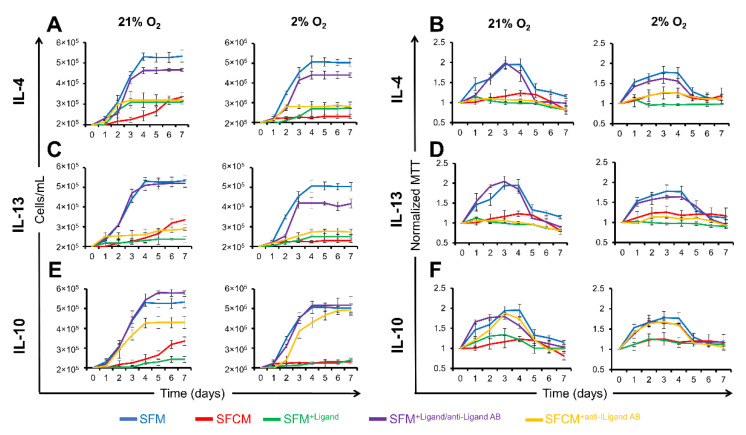
Proliferation of non-polarised Jurkat T cells is restored in IL-10-devoid SFCM. Growth curve plots calculated from cell counts of non-polarised Jurkat T cells cultured in SFM, SFCM, SFM^+Ligand^, SFM^+Ligand/antiLigand AB^, and SFCM^+antiLigand AB^ in air (21% O_2_) and physoxia (2% O_2_) environments over a 7 day period. (**A**) IL-4, (**C**) IL-13, and (**E**) IL-10. Data expressed as mean ± SD (*n* = 3). Optical density (O.D.) values for MTT assays of non-polarised Jurkat T cells cultured in SFM, SFCM, SFM^+Ligand^, SFM^+Ligand/antiLigand AB^, and SFCM^+antiLigand AB^ in both air (21% O_2_) and physoxia (2% O_2_) environments over a 7 day period. (**B**) IL-4, (**D**) IL-13, and (**F**) IL-10. MTT assay values at each time point were normalised to day 0. Data expressed as mean ± SD, (*n* = 3).

In SFM, the cell growth curve followed the anticipated pattern of lag, log, and achieved the stationary phase at day 4 (21% O_2_ = 5.3 × 10^5^ ± 1.8 × 10^4^ cells/mL and 2% O_2_ = 5.1 × 10^5^ ± 3.1 × 10^4^ cells/mL), whereas the maximal growth curve in SFCM was significantly lower than that of SFM with maximum differences achieved at the stationary phase at day 4 (21% O_2_ = 2.4 × 10^5^ ± 2.5 × 10^4^ cells/mL and 2% O_2_ = 2.2 × 10^5^ ± 5.8 × 10^3^ cells/mL) (Figure 3). In SFM, the metabolism increased gradually to a maximum at day 4 (21% O_2_ = 2.0 ± 0.1 O.D. and 2% O_2_ = 1.8 ± 0.1 O.D.), whereas metabolic rates in SFCM were significantly lower than SFM at all time points, day 4 (21% O_2_ = 1.2 ± 0.1 O.D. and 2% O_2_ = 1.2 ± 0.1 O.D.) (Figure 3).

To identify specific biomolecule(s) responsible for this phenotype in SFCM, cytokines with prominent anti-inflammatory properties, which we previously confirmed as being components of SFCM, were tested, including IL-4, IL-10, or IL-13 [15]. Cytokines individually induced the suppression of proliferation in non-polarised Jurkat T cells in both normoxia and physoxia environments at day 4 [(SFM/SFM^+IL4^; ~2 fold reduction), (SFM/SFM^+IL10^; >2 fold reduction), and (SFM/SFM^+IL13^; >2 fold reduction)] (Figure 3A,C,E). In addition, the metabolism was suppressed through the addition of cytokines [(SFM/SFM^+IL−4^; ~2-fold reduction) (Figure 3A,B), (SFM/SFM^+IL−13^; ~2-fold reduction)] (Figure 3C,D), and (SFM/SFM^+IL−10^; ~2-fold reduction) (Figure 3B,D,F).

IL-10 neutralisation in SFCM induced restoration of proliferation in both normoxia and physoxia environments at day 4 (SFM/SFCM^+anti-IL−10^; ~1-fold increase). Conversely, IL-4/IL-13 neutralisation was associated with no restoration of proliferation in either normoxia or physoxia environments at day 4 [(SFM/SFM^+antiIL−4^; ~2-fold reduction), and (SFM/SFCM^+anti-IL−13^; ~2-fold reduction)]. Collectively, this suggests that IL-10 in SFCM is associated with the suppression of proliferation in non-polarised Jurkat T cells, irrespective of IL-4 and IL-13 activity. 

#### 2.3.2. IL-10 Suppresses the Proliferation of Polarised Jurkat T Cells

**Figure 4:**
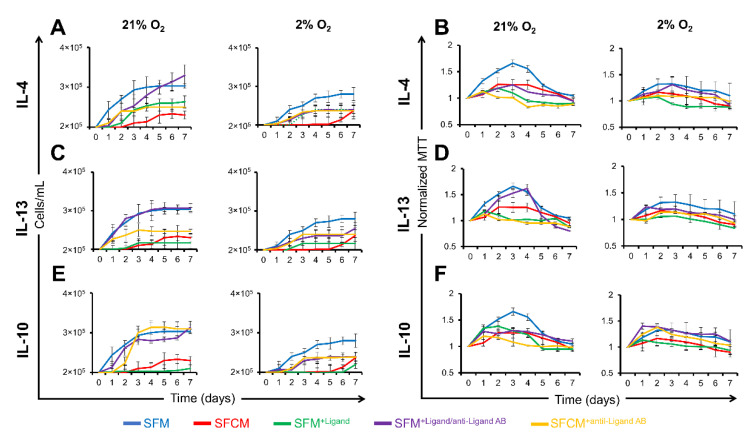
Proliferation of polarised Jurkat T cells is restored in IL-10-devoid SFCM. Growth curve plots calculated from cell counts of polarised Jurkat T cells cultured in SFM, SFCM, SFM^+Ligand^, SFM^+Ligand/antiLigand AB^, and SFCM^+antiLigand AB^ in normoxia (21% O_2_) and hypoxia (2% O_2_) environments over a 7 day period. (**A**) IL-4, (**C**) IL-13, and (**E**) IL-10. Data expressed as mean ± SD (*n* = 3). Optical density (O.D.) values for MTT assays of non-polarised Jurkat T cells cultured in SFM, SFCM, SFM^+Ligand^, SFM^+Ligand/antiLigand AB^, and SFCM^+antiLigand AB^ in both air (21% O_2_) and physoxia (2% O_2_) environments over a 7 day period. (**B**) IL-4, (**D**) IL-13, and (**F**) IL-10. MTT assay values at each time point were normalised to day 0. Data expressed as mean ± SD, (*n* = 3).

Cell counts following activation revealed similar patterns of proliferation suppression in both SFCM and SFM^+ligand^ (IL-4, IL-13, and IL-10) compared to SFM and SFM^+Ligand+anti-Ligand^ (IL-4, IL-13, and IL-10) with a slight restoration (~1 fold) achieved with the neutralisation of either anti-inflammatory cytokines (Figure 4A,C,E). Similar patterns in the reduction in MTT values were noted in both SFCM or SFM^+Ligand^ cells compared to SFM or SFM^+Ligand+anti-Ligand^ with no restoration achieved following neutralisation of either anti-inflammatory cytokine (Figure 4B,D,F).

**Figure 5:**
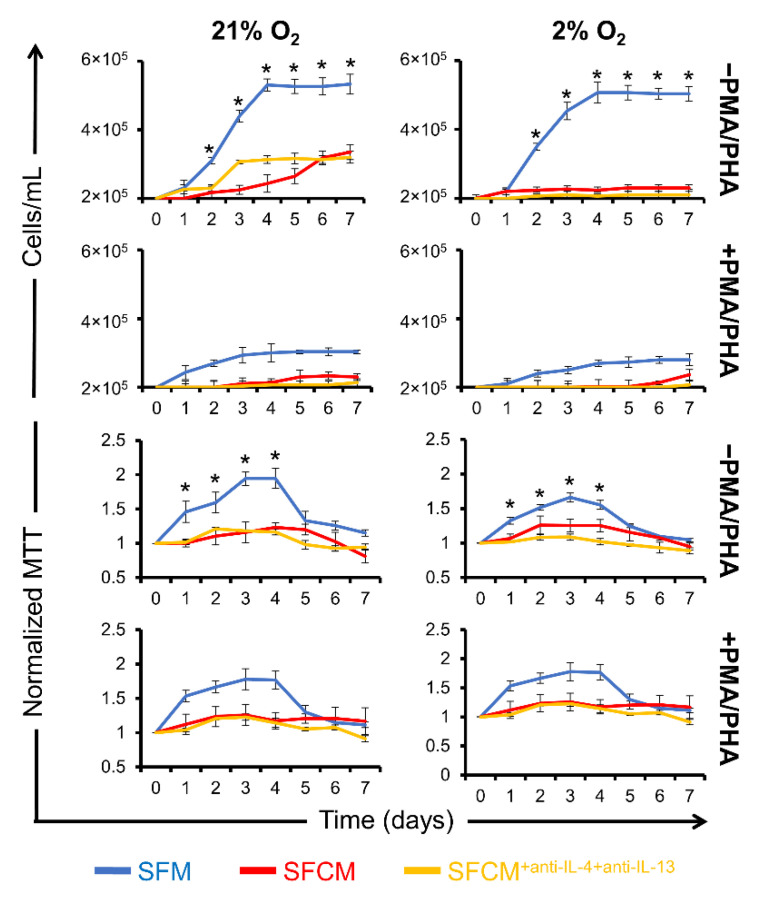
IL-4/IL-13-devoid SFCM failed to restore proliferation. Growth curves plotted from cell numbers (cells/mL) and optical density (O.D.) values for MTT assays for polarised and non-polarised Jurkat T cells in SFCM^+antiIL−4+antiIL−13^ compared to SFCM in air (21% O_2_) and physoxia (2% O_2_) environments over 7 days. Growth curves were obtained by haemocytometer-based cell counting. Data expressed as mean ± SD (*n* = 3). MTT assay values at each time point were normalised to day 0. Data expressed as mean ± SD, (*n* = 3), * *p* < 0.05.

To confirm the biomolecule(s) responsible for the suppression of proliferation in polarised Jurkat T cells exposed to SFCM further, these cytokines were again individually blocked by their specific polyclonal antibodies. IL-10 neutralisation in SFCM induced restoration of proliferation in both air and physoxia environments, day 4 (SFM/SFCM^+anti-IL−10^; ~1-fold increase) (Figure 4E). Conversely, IL-4/IL-13 neutralisation was associated with no restoration of proliferation in either normoxia or physoxia environments, day 4 [(SFM/SFCM^+anti-IL−4^; ~2-fold decrease) and (SFM/SFCM^+anti-IL−13^; ~2-fold decrease)] (Figure 5). MTT assays showed that polarised Jurkat T cells displayed a reduced metabolism compared to non-polarised cells. Similar patterns of the suppression of metabolism were noted in both SFCM or SFM^+Ligand^ cells compared to SFM or SFM^+Ligand+antiLigand^ with no restoration (~1 fold) achieved following neutralisation of either anti-inflammatory cytokines. Taken together, this suggests that IL-10 in SFCM is associated with the suppression of proliferation in polarised Jurkat T cells and occurs irrespective of IL-4 and IL-13 activity. 

**Figure 6:**
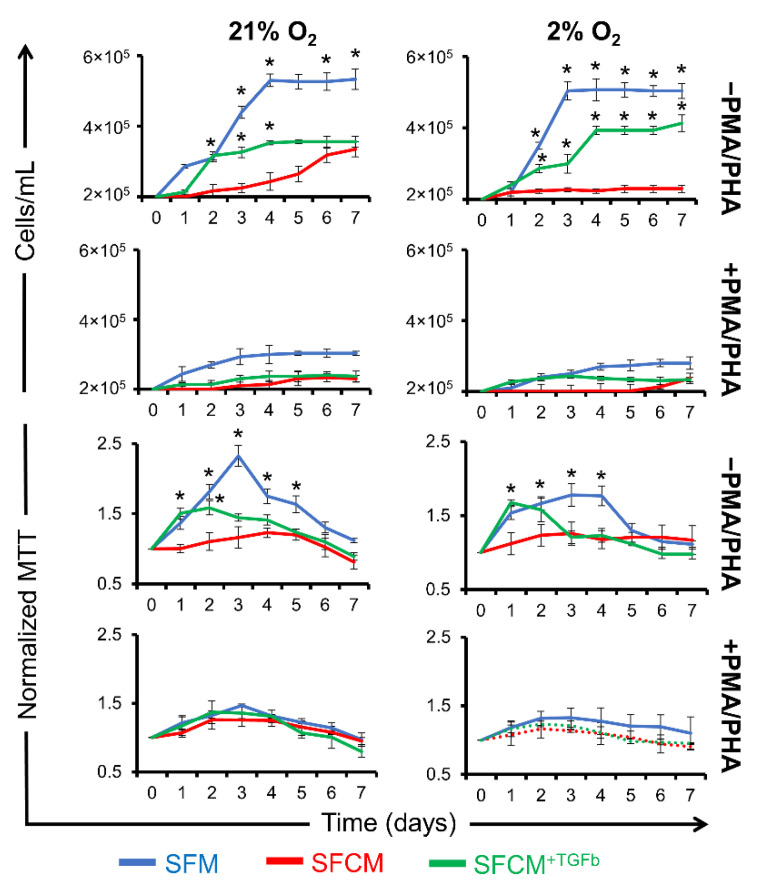
TGFb failed to suppress Jurkat cell proliferation. Growth curves plotted from cell numbers (cells/mL) and optical density (O.D.) values for MTT assays for polarised Jurkat T cells cultured in SFM, SFCM, SFM^+Ligand^, SFM^+Ligand/antiLigand AB^, and SFCM^+antiLigand AB^ in air (21% O_2_) and physoxia (2% O_2_) environments over a 7 day period. Growth curves were obtained by haemocytometer-based cell counting, and the results were confirmed through MTT assays. Data expressed as mean ± SD; each result represents a replicate of 3 independent experiments (*n* = 3). One-way ANOVA was conducted with Tukey’s test to determine pairwise significant difference. * *p* < 0.001.

Further, TGFb did not inhibit the proliferation of non-polarised Jurkat T cells in SFM, indicating that activation of the T cell model is mainly linked to the presence or absence of IL-10 in SFCM, day 4 (SFM/SFM^+TGFb^; ~1-fold decrease) (Figure 6).

#### 2.3.3. IL-10 Suppresses IL-2 Secretion

**Figure 7:**
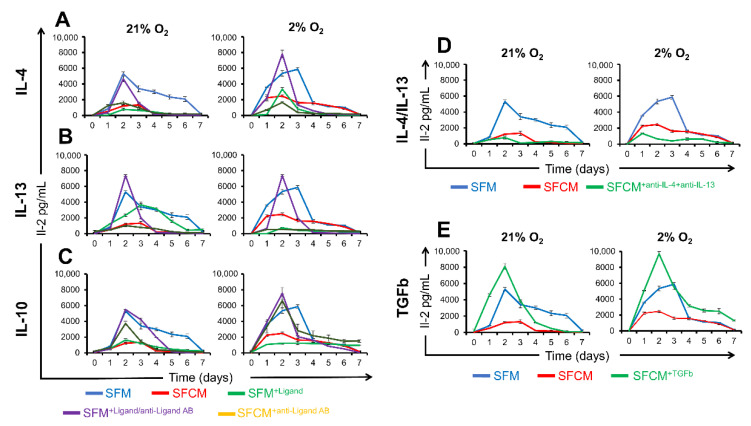
IL-10-devoid SFCM restores IL-2 secretion in activated Jurkat T cells. ELISA-based IL-2 detection (pg/mL) of Jurkat T cell culture media following in vitro activation over 7-days. PMA/PHA activated Jurkat T cells were cultured in different conditions consisting of SFM, SFCM, SFM^+Ligand^, SFM^+Ligand/antiLigand AB^, and SFCM^+antiLigand AB^ in both air (21% O_2_) and physoxia (2% O_2_) oxygen environments. (**A**) IL-4 studies, (**B**) IL-13 studies, and (**C**) IL-10 studies. (**D**) ELISA-based IL-2 detection assay for the media of activated Jurkat cells cultured in SFM, SFCM, and SFM^+antiIL−4/antiIL−13^ in both air and physoxia oxygen tensions. (**E**) ELISA-based IL-2 detection assay for the media of activated Jurkat cells cultured in SFM, SFCM, SFCM^+TGFb^. Data expressed as mean ± SD; each result represents a replicate of 3 independent experiments (*n* = 3).

In vivo T cell stimulation occurs following antigen presentation on the surface of antigen presenting cells and is associated with IL-2 production where subsequent proliferation leads to the launching of the immune response. In contrast, in vitro polarisation of Jurkat T cells by PMA/PHA results in an abrogation of proliferation and the production/release of IL-2. We previously demonstrated that the IL-2 release from polarised Jurkat cells was equivalent in both normoxia and physoxia (Figure 1D) and now sought to explore if SFCM impacted its release following activation. We noted the IL-2 release following PMA/PHA treatment over 7 days which reached a maximum in SFM at day 2 [21% O_2_ (5305 ± 211 pg/mL) and 2% O_2_ (5347 ± 327 pg/mL)] and declined thereafter reaching a minimum at day 7 [21% O_2_ (258 ± 75 pg/mL) and 2% O_2_ (145 ± 32 pg/mL)] (Figure 7). SFCM suppressed IL-2 secretion in polarised Jurkat T cells in both normoxia and physoxia, day 2 [21% O_2_ (2461 ± 178 pg/mL) and 2% O_2_ (1625 ± 159 pg/mL)]. Following our earlier observations, we next explored whether IL-10, IL-4, and IL-13 displayed a capacity to block the IL-2 release. Cytokines added individually led to a reduction in IL-2 secretion in polarised T cells in a SFCM-comparable manner in both normoxia and physoxia environments, day 2 [(SFM/SFM^+IL−4^; >2-fold reduction) (Figure 7A), (SFM/SFM^+IL−13^; >2-fold reduction)] (Figure 7B) and (SFM/SFM^+IL−10^; >2-fold reduction) (Figure 7C). In addition, neutralisation of these cytokines via ligand specific polyclonal antibodies resulted in restoration of IL-2 secretion in SFM, day 2 [(SFM^+IL−4^/SFM^+IL−4+anti-IL−4^; <2-fold increase), (SFM^+IL−10^/SFM^+IL−10+anti-IL−10^; <2-fold increase), and (SFM/SFCM^+anti-IL−13^; <2-fold increase)]. This suggests that IL-10 in SFCM is associated with the suppression of IL-2 secretion regardless of the presence or absence of both IL-4/IL-13. To address IL-4/IL-13 polymorphism, we simultaneously blocked IL-4 and IL-13 from SFCM with their specific polyclonal antibodies. This again resulted in a failure to reverse the blockage of IL-2 secretion, day 2 (SFM/SFCM^anti-IL−4/anti-IL−13^; ~8 fold) (Figure 7D). 

Additionally, TGFb failed to inhibit IL-2 secretion by polarised Jurkat T cells in SFM, day 2 (SFM/SFM^+TGFb^ < 2 fold), indicating that activation of the T cell was associated with IL-10 status (Figure 7E). These results indicate that Jurkat T-cell polarisation in SFCM is linked to IL-10 ligands highlighting the importance of IL-10 post-receptor signalling pathways.

## 3. Discussion

The application of hMSCs in clinical settings is an attractive prospect [41,42,43]. Many clinical trials to date focused on immune-mediated diseases including Crohn’s disease, diabetes mellitus, graft-versus-host disease (GvHD), hepatitis, and rheumatoid arthritis [8,44,45,46,47]. However, their first pass pulmonary engraftment potential makes the mode of action questionable [48,49]. Recent in vitro and in vivo studies confirmed that the regenerative mode of action of hMSCs is partially linked to the release of trophic factors rather than their functional incorporation [50,51,52,53]. However, the released trophic factors are a mixture of biomolecules with various functions, including pro-inflammatory, anti-inflammatory, pleiotropic cytokines, chemokines, growth factors, and angiogenic factors [15,54,55,56]. The concentration of these cytokines varies depending on the source of hMSCs, their isolation, and the culture protocol [56,57,58]. Riberio et al. (2012) compared the composition of conditioned media collected from either adipose or umbilical-cord-derived stem cells, demonstrating an altered secretome profile and consequently differential effects on metabolic viability when applied to neuronal cell cultures [56]. The present report describes the mode of action of the hMSC secretome by testing the efficacy of SFCM itself and a selection of its components: IL-4, IL-10, IL-13, and TGFb anti-inflammatory cytokines on the model Jurkat T cell line. 

Oxygen concentration is an important factor for both hMSC culture and in vitro immune response models. Physoxia is a characteristic feature of hMSC niches, which are under continuous oxygen gradient exposure depending on the local tissue microenvironment, such as bone marrow (1–6% O_2_), adipose tissue (2–8% O_2_), and neural tissues (1–8% O_2_) [59]. The oxygen tension is higher in most endogenous tissue compartments (4–14% O_2_) but is still less than that of ambient 21% air oxygen [33]. Additionally, the inflammation zone is under pathological hypoxia due to activation of coagulation cascades leading to the activation of immune cells [60]. In this study, SFCM was collected in both 21% O_2_ and 2% O_2_ environments to simulate oxygen concentrations present in the in vivo bone marrow niches and help create a physiologically relevant in vitro immune response model. Various in vitro and in vivo studies as well as our previously published studies reported the importance of 2% O_2_ collected SFCM in comparison to air oxygen SFCM [15,61,62]. 

Exposure of Jurkat T cells to PMA/PHA in this study resulted in their activation and indicative IL-2 secretion, abrogation of proliferation, and increases in the cell surface area. This is in line with previously published reports that T cell line exposure to mitogenic stimuli results in their differentiation and suppression of proliferation [39]. Non-stimulated Jurkat T cells displayed normal proliferation passing through the lag, log, and stationary phases whilst, upon activation, the cells reached a plateau after the lag phase showing no log or stationary phases. 

It was reported that the immunosuppressive activity of MSCs is mainly mediated through soluble factors [63]. However, there is lack of clarity surrounding the specific signalling biomolecules responsible for immunosuppression, making the exact mechanism of action questionable [64,65]. The secretome profile is a mixture of complex protein-based bioactive factors, including the stem cell factor (SCF), IL-6, IL-8, IL-10, IL-12, IFNγ, prostaglandin E2 (PGE2), vascular endothelial growth factor (VEGF), macrophage colony stimulating factor (M-CSF), hepatocyte growth factor (HGF), and transforming growth factor -b1 (TGFb1) [11,55,58,66]. Di Nicola et al. confirmed that the in vitro immunosuppressive activity of MSCs could be reverted through blocking the effects of both TGFb and HGF [4]. However, neutralisation of either TGFb or HGF individually resulted in minimal restoration of the immune response, while their addition in combination achieved a comparable immunosuppression to MSCs [67]. Failure to achieve immunosuppression with TGFb alone was reported by different in vitro studies [68,69,70]. Mori et al. concluded that TGFb mediates its action synergistically with HGF through JNK-dependent Smad2/3 phosphorylation at their promotor regions [71]. In the present study, TGFb failed to induce immunosuppression, confirming that TGFb has no role in SFCM-mediated immunosuppression. Jurkat T cells were proliferative in SFM^+TGFb^ and produced IL-2 following PMA/PHA activation, confirming that TGFb alone has no immunosuppressive properties. Interestingly, cyclosporine A, a potent immunosuppressive agent, was previously associated with elevated levels of intracellular TGFb and its receptor, suggesting that its mechanism of action is mainly linked to TGFb [72]. These studies indicate that there is a relationship between the mode of action of TGFb and HGF and could explain the failure to respond to SFM^+TGFb^ in the present study.

Naïve T cell proliferation is regulated by IL-2 and CD25 (IL-2 receptor alpha subunit (IL-2Ra)), whilst their maturation is under the control of different cytokines, including IL-2, IL-12, and IFNγ which promote Th1 differentiation and IL-4, IL-5, IL-9, IL-10, and IL-13 which promote Th2 differentiation [73]. Hence, IL-4 and IL-13 are part of the differentiation mechanism of T cells, sharing 25% structural similarity, characterised by receptor overlapping phenomena via sharing the receptor subunit (IL-4Rα) for their signal transduction; therefore, IL-13 can induce many functional properties of IL-4 [73,74]. In the present study, the immunosuppression achieved by either IL-4 or IL-13, when individually tested on Jurkat T cells, was confirmed by the suppression of proliferation and reduction in IL-2 production. However, neutralisation of IL-4/IL-13 individually, or in combination, induced no restoration in the immunosuppressive activity of SFCM, suggesting that alternative pathways could be responsible for the immunomodulation achieved by SFCM. Dupilumab, a human monoclonal antibody for the treatment of asthmatic patients, targets both IL-4 and IL-13; the mechanism of this antibody is based on the inhibition of IL-4/IL-13 engagement with the α subunit of the IL-4 receptor [75]. Despite successful immunosuppression achieved by dupilumab and the failure to achieve immune response restoration following IL-4/IL-13 neutralisation in SFCM, their participation in immunosuppression is not negligible, indicated by the suppression of proliferation and revoked IL-2 secretion by Jurkat T cells. Cytokine cross-reactivity and receptor affinity are likely responsible, noting that SFCM is a mixture of different cytokines, and most cytokines which are present in SFCM, such as, IL-2, IL-4, IL-7, IL-9, IL-15, and IL-21, show IL-4 receptor sharing [73].

IL-4/IL-13 share post-receptor latent proteins and the translation pathway (STAT6), and the translocation of this second messenger protein to the nucleus results in the transcription of anti-inflammatory genes [73,74]; moreover, pharmacological targeting of either cytokine alone achieved limited therapeutic activity in comparison to combined therapy [73]. However, IL-10 mediated its immunosuppressive activity through a distinct (STAT3) post-receptor translation pathway [23]. Various reports suggest that IL-10 has a unique capacity to block the synthesis of proinflammatory cytokines, including TNFa, IFNγ, IL-1B, and IL-6 [23,24]. Moreover, IL-10 post-receptor translation pathways involve the induction of more than one post-receptor translation pathway including STAT1, STAT3, and STAT5. STAT1 and STAT5 are not involved directly in IL-10 receptor stimulation; however, their knockout is associated with the modulation of the cellular response to IL-10 [23]. These results are conflicting, and clarification is required to dissect the immunosuppression activity of SFCM through IL-10 receptor blocking or JAK1 knockout rather than simple polyclonal antibody neutralisation.

The critical role of the potent anti-inflammatory cytokine IL-10 during host infection involves the modulation of both innate and adaptive immunity through the suppression of T cells, NK, and macrophages, helping to protect from the effects of excessive and prolonged inflammation [23,76,77,78,79]. Conversely, persistent viral infection results in the upregulation of IL-10, leading to impairment of the T cell response and a lack of efficient clearance of pathogens from the host [80]. Regulation of IL-10 and the effects on T cell homeostasis are therefore critical to the proper functioning of the immune response. Previous studies show that rheumatoid arthritis patients display lower serum levels of IL-10 compared to healthy patients [81]. In addition, IL-10 secreting MSCs demonstrated enhanced cell survival and therapeutic benefits in models for Duchenne muscular dystrophy (DMD) [82]. The ability of MSCs to migrate, accumulate, and exhibit both immunosuppressant and antiapoptotic effects through the secretion of IL-10 at sites of injury makes them an attractive prospect in both cell and cell-free therapies for diseases with chronic inflammatory pathologies [82,83]. 

## 4. Materials and Methods

### 4.1. Cell Line Culture

#### 4.1.1. hMSCs

hMSCs were isolated and expanded from human bone marrow aspirate (BMA) using an adherence-based methodology [35]. Three donor human BMAs (two male and one female, ages 20–36) were purchased from Lonza, USA and seeded at a density of 1 × 10^5^ mononuclear cells/cm^2^ on fibronectin pre-coated culture flasks in Dulbecco’s Modified Eagle Medium (DMEM) media supplemented with 5% (*v*/*v*) fetal bovine serum (FBS), 1% (*v*/*v*) L-glutamine, 1% (*v*/*v*) non-essential amino acids (NEAA), and 1% (*v*/*v*) Penicillin-Streptomycin-Amphotericin B (PSA) (Lonza, Slough, UK). Seeded flasks were incubated in humidified incubators with distinct oxygen tensions of 21% O_2_ or 2% O_2_. After 7 days, half of the media volume was removed and replaced with fresh antibiotic-free growth medium followed by a complete media change after a further 7 days. Media was then changed every 3 days until confluent. Once confluent, hMSC were enzymatically passaged with 1% Trypsin/EDTA (Lonza, Slough, UK) at 1:2 split ratios. Passage 1 cells and their CM were used for all experiments.

SFCM was produced by washing 70% confluent hMSC seeded T75 flasks with phosphate-buffered saline (PBS) followed by the addition of 15 mL serum-free media (SFM) consisting of DMEM supplemented with 1% L-glutamine (*v*/*v*) and 1% NEAA (*v*/*v*). For conditioning, 20 mL of SFM were added to hMSC cultures and incubated for 24 h. Conditioned media was then collected, centrifuged for 10 min at 300× *g*, and stored at −80 °C as SFCM. Prior to use, SFCM was thawed and filtered (0.2 μm). All SFCM was produced from hMSC cultures at passage 1. Time taken for hMSC isolation from BMA plating, expansion, passaging, and confluency at passage 1 was 28–29 days (28 days for donors 1 and 2, 29 days for donor 3) and was consistent between the oxygen concentrations. 

Positive expression for CD73, CD90, and CD105 and negligible expression for CD14, CD19, CD34, CD45, and HLA-DR surface molecules were confirmed, as well as trilineage differentiation potential according to ISCT guidelines, and were published previously [15]. 

#### 4.1.2. Jurkat T Cells

The Jurkat cell line (ATCC clone E6-1) was cultured in suspension in tissue culture flasks. Cells were passaged by centrifuging at 180× *g* for 3 min; the supernatant was removed and the cell pellet re-suspended in fresh growth media (GM) consisting of Roswell Park Memorial Institute (RPMI)-1640 (Lonza, Slough UK) supplemented with 10% (*v*/*v*) FBS, 1% L-glutamine, and 1% NEAA. Cells were passaged and seeded at a density of 1 × 10^5^ cells/mL with media changes performed twice per week. Seeded flasks were incubated in humidified incubators with distinct oxygen tensions of either 21% O_2_ or 2% O_2_.

#### 4.1.3. Jurkat Cell Activation

Jurkat cells were seeded at 5 × 10^5^ cells/mL for activation through the addition of 50 ng of Phorbol Myristate Acetate (PMA) (Sigma-Aldrich, Gillingham, UK) and 1 µg of Phytohaemaglutinin (PHA) (Sigma-Aldrich, Gillingham, UK) to the cell culture media. The activation was performed for a period of 24 h in a humidified incubator at 37 °C at either 21% O_2_ or 2% O_2_.

### 4.2. Cell Viability and Proliferation Assays

#### 4.2.1. Cell Counting

Haemocytometer cell counts were performed on time-point samples over 7 days to determine the rate of cell growth from an initial seeding density of 2 × 10^5^ cells/mL. Cell counts were performed on the cells in different conditions, including, activated and inactivated cells in their GM, SFM, and SFCM, and under different oxygen tensions, 2% O_2_ and 21% O_2_. Counts were performed on triplicate flasks. 

#### 4.2.2. MTT Assay

The MTT (3-[4,5-dimethylthiazol-2-yl]-2,5 diphenyl tetrazolium bromide) assay (Sigma-Aldrich, Gillingham, UK) was used to determine metabolic activity. Jurkat cells were seeded in triplicate at a concentration of 2 × 10^5^ cells/mL before incubation in MTT for 4 h at 37 °C as per manufacturer’s instructions. A total of 50 µL of DMSO were added to each well to dissolve formazan crystals, and the plate was incubated again for 45 min. The optical density (O.D.) of each sample was determined by reading at 570 nm on a Synergy2 plate reader (BioTek, Cheadle, UK). 

#### 4.2.3. Cytospin

The Cytospin technique was used to assess Jurkat cell morphology. To prepare Cytospin slides for imaging, 5 × 10^4^ cells were centrifuged and the supernatant removed, and pellets were washed twice in cold PBS and resuspended in 1 mL PBS. The cells were then pipetted into the Cytofunnel (Fisher Scientific, Loughborough, UK) and centrifuged by Cytospin-centrifuge (Fisher Scientific, Loughborough, UK) at 300× *g* for 2 min. Slides were then removed and air dried for 15 min and fixed with 95% ethanol for 15 min. Following air drying, the slides were stained with May–Grünwald solution (Sigma-Aldrich, Gillingham, UK) for 5 min, washed, and further staining with Giemsa solution (Sigma-Aldrich, Gillingham, UK) for 15 min. A xylene mounting agent was placed over the slides to fix the cover slides. Images were captured, and the cell surface area was calculated for 100 cells using ImageJ software (NIH, Maryland, MD, USA). 

### 4.3. Cytokine Challenging

To identify if the immunosuppression by SFCM was cytokine-driven, four anti-inflammatory targets were chosen: IL-4, IL-10, IL-13, and TGFb. These cytokines were individually tested on the Jurkat cell line models in SFM, and a cytomix is considered to account for the IL-4/IL-13 receptor overlapping phenomena. Concentrations were identified via dose-response curves via testing a serial dilution of an individual cytokine in SFM on the Jurkat cell line models: IL-4 (PeproTech, London, UK #200-04), IL-10 (PeproTech, London, UK #200-10), IL-13 (PeproTech, London, UK #200-04), and TGFb (PeproTech, London, UK #100-36E). MTT was conducted after 24 h of cell exposure to a serial dose of IL-4 (0–10,000 pg/mL), IL-13 (0–10,000 pg/mL), IL-10 (0–50 ng/mL), and TGFb (0–100 ng/mL). Toxic doses were ignored, and the highest non-toxic doses of each [(IL-4 and IL-13 (250 pg/mL), IL-10 (1 ng/mL), and TGFb (5 ng/mL)] were considered as target doses.

### 4.4. Cytokine Blocking

Cytokines were either individually or in combination (IL-4/IL-13) blocked from SFCM. These cytokines were blocked in SFCM using an excess amount of rabbit polyclonal antibodies: IL-4 (PeproTech, London, UK #500-P24), IL-10 (PeproTech, London, UK #500-P20), and IL-13 (PeproTech, London, UK #500-P13). To neutralise these cytokines from SFCM, IL-10 was neutralised by 2 μg/mL of anti-human IL-10, IL-4 neutralised by 100 ng/mL anti-human IL-4, and IL-13 neutralised by 200 ng/mL anti-human IL-13.

### 4.5. ELISA Assay

ELISA assays were conducted for the detection of IL-2 (PeproTech, London, UK #900-K12), IL-4 (PeproTech, London, UK #900-M14), and IL-10 (PeproTech, London, UK #900-M21). Standard serial dilutions and culture media samples were loaded in triplicate into an overnight pre-coated surface with cytokine specific capture antibodies. Blocking was carried out for 1 h using 1% BSA-blocking buffer, followed by a 2 h incubation with a diluted detection antibody mixture and 30 min with a diluted avidin-HRP conjugate. Each step was accompanied by forcibly discarding the contents and four washing steps with diluted detergent buffer. Finally, an enzymatic reaction initiated by the addition of an ABTS-substrate (2,2′-Azino-bis (3-ethylbenzothiazoline-6- sulfonic acid)), leading to a bluish-green colour development within 5–15 min during which a visible signal was detected at 405 nm via a plate reader. The concentrations of unknown samples were determined by the interpolation of the standard calibration curves for each component.

### 4.6. Statistical Analysis

Statistical analysis was conducted between SFCM and SFM using a two-sample t-test for most of the measured parameters. For comparison of more than 2 groups, a one-way ANOVA with Tukey’s multiple comparisons test was performed to determine pair-wise statistical significance; *p* ≤ 0.05 was considered significant. The analysis was performed using GraphPad Prism 6 (San Diego, CA, USA). Unless otherwise stated, all values quoted in the results are mean ± standard deviation (SD).

## 5. Conclusions

Collectively, the present findings support the suggestion that IL-10 in hMSC SFCM plays an immunosuppressive role in Jurkat T cell proliferation and activation. The specificity of IL-10 in this role was confirmed through the inability of IL-4 and IL-13 neutralisation in SFCM to restore Jurkat cell proliferation. Further, it was demonstrated that TGFb has no role in SFCM-mediated immunosuppression. The identification of specific immunosuppressive components of the hMSC secretome and the development of a mechanistic understanding of their action pave the way for the development of more controlled cell-free therapies in the future, expanding the treatment options available for patients suffering from diseases with an inflammatory component.

## Data Availability

Not applicable.

## Supplementary Materials

The following supporting information can be downloaded at: https://www.mdpi.com/article/10.3390/ijms232113596/s1.
