# Supplementary figures and images for: Human Mesenchymal Stem Cell Secretome Driven T Cell Immunomodulation Is IL-10 Dependent
Source: Int J Mol Sci. 2022 Nov 6;23(21):13596. doi: 10.3390/ijms232113596 (PMC9658100; doi:10.3390/ijms232113596)

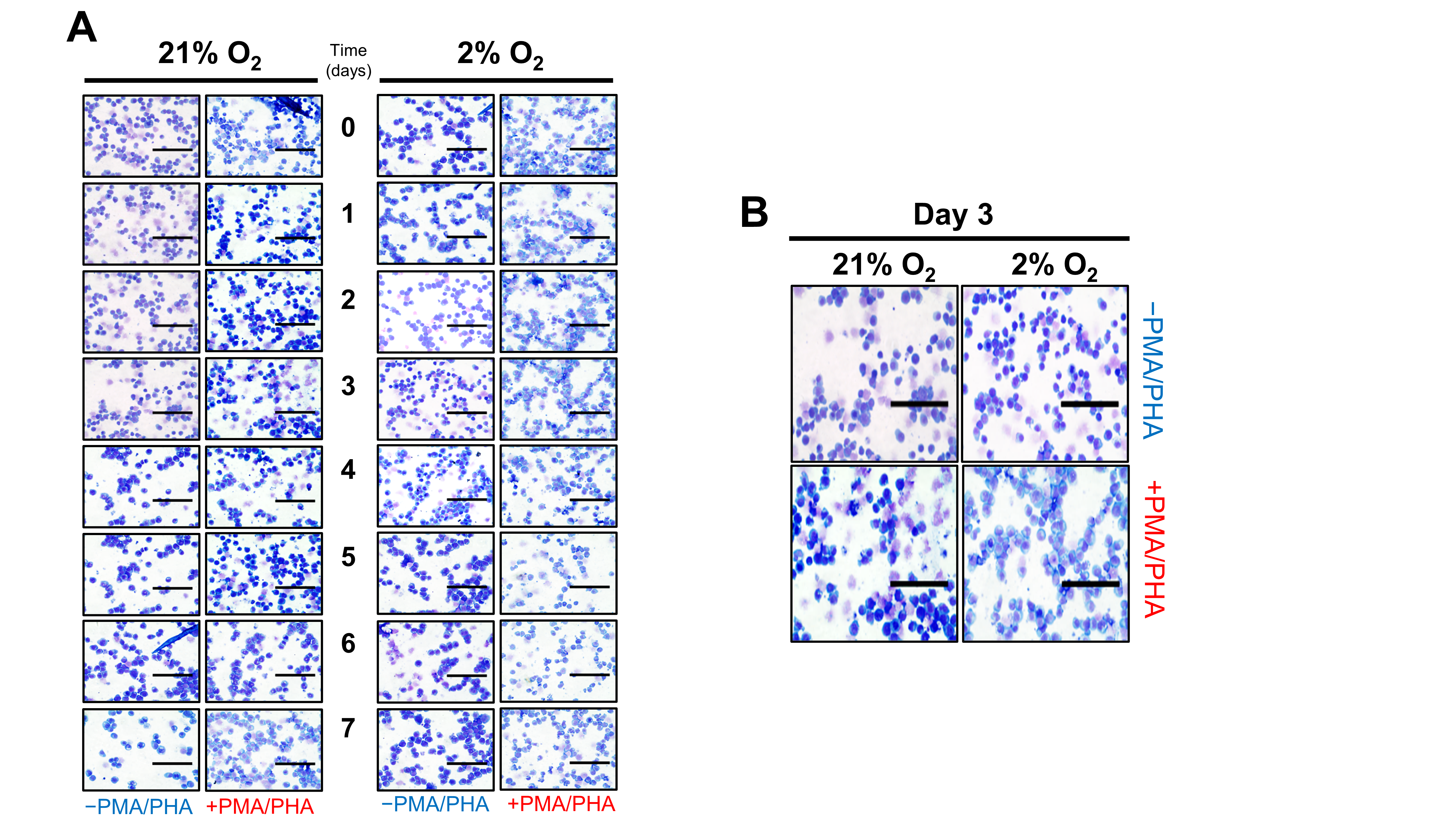

Supplement: Supplementary file 1 [file ijms-23-13596-s001.zip › ijms-2010154-supplementary.tif]
